# Supplementary material for: Awareness and perceptions of Long COVID among people in the REACT programme: Early insights from a pilot interview study
Source: PLoS One. 2023 Jan 26;18(1):e0280943. doi: 10.1371/journal.pone.0280943 (PMC9879384; doi:10.1371/journal.pone.0280943)
Supplement: S1 Appendix — (DOCX) [file pone.0280943.s002.docx]

**S1 Appendix 1. Topic Guide**

**Experiences of Covid**

To explore the experiences and journeys of people with Covid

How to use this guide

*Italics are interviewer instructions*

1. **Topic/Question (preface with ‘can you tell me about’ or similar)**

- = Probe (to gain more detail from participants answers using their words or ideas)
  - = Prompt (newly introduced examples or questions if participant isn’t able to answer or gives yes/no)

*Remind participants to only answer questions they feel comfortable with*

1. **Background** *Explore participants background*

**Can you tell me a little bit of general background about yourself?**

- Living situation
- Employment status/field of work (prior to pandemic)
  - Key worker (e.g. NHS staff, teacher etc.)

**Can you tell me about your health prior to the pandemic?**

- General view of health and lifestyle
- Any pre-existing health conditions (ongoing)
- Any prior illnesses
- Any prior unexplained/undiagnosed symptoms

**2. Initial experience of COVID-19**

**I understand that you were diagnosed with COVID-19. Can you describe to me what happened initially?**

- When did you suspect you had Covid-19?
- Was testing available?
- Were you tested within the first 5 days of symptoms?
- Where/ how did you get tested?
- What symptoms did you experience?
  - Type (Cough, breathlessness, loss of taste or smell, fatigue, fever…)
  - Severity
  - Duration
- How did the illness progress?
- Can you tell me about any medical help you sought?
  - Medical advice: 111/pharmacy/GP/999/hospital/other
- Did you know other people (family, friends, colleagues) who were also affected?

**3. Ongoing journey**

**What happened after your initial illness/ diagnosis?**

- How long did your initial symptoms last? Did they end? Did they change?
  - Fluctuations in existing symptoms
    - *Probe for each symptom mentioned*
  - Different or new symptoms
- What were your thoughts/feelings about these symptoms at the time?
- What help, if any, did you get for ongoing symptoms?
  - GP, hospital, investigations

**When did you first become aware of Long Covid?**

- How long were you experiencing symptoms for before this happened?
- Do you think that you had/have Long Covid?

Have you been diagnosed with Long Covid?

- How does this make you feel?

**4. Long Covid**

**Can you tell me a bit more about your experience of Long Covid and this affected you?**

- How have you felt physically?
- How have you felt emotionally/mentally?
- Can you describe how different areas of your life have been impacted?
  - Physical activities: walking short or long distances/climbing stairs/ standing/sitting
  - Daily life: Cooking/cleaning/personal care/entertainment
  - Activities: Work/education/socialising/exercise/hobbies/family life and relationships
- Can you describe how your wellbeing has been affected?

**Did you try to seek any medical help or treatment, what was this like?**

- Did you find it easy or difficult to access help or treatment?
- Do you remember any particular conversations you had?
- How did you find talking to someone about your symptoms?
- Was this helpful?

**Did you try to seek any other kind of support?**

**Can you tell me about any Long Covid support groups or communities you may have joined?**

- What groups did you join?
  - Online: Body politic slack/Long Covid facebook group/twitter
  - Offline support: Community support/local groups
- What prompted you to join/not to join them?
- What sort of support/information do they provide?
- How do you feel about the group(s) now compared to when you first joined?

**5. Current situation**

**Thinking about your current symptoms, day to day…**

- Is any one symptom bothering you more than the others at the moment?
  - Is this always the case?
- Do your symptoms fluctuate/change day to day?
- If so, can you describe to me to me what a ‘good’ or ‘bad’ day looks like

**Have you experienced any longer term changes in your symptoms? Can you tell me a bit about this?**

- When did you notice things were changing?
- Did things improve or get worse?
- How quickly did these changes occur?
- How did this make you feel?

**Do you think anything in particular has helped with your recovery?**

- Your living situation/work/lifestyle/prior health condition
- Any treatment/medication?
- Any activities or plans you are following?

**Do you think anything in particular has impeded your recovery?**

- Your living situation/work/lifestyle/prior health condition
- Any treatment/medication?
- Any activities or plans you are following?

**6. What services do you think would be/ would have been helpful for you and others with Long Covid?**

- Who should provide these services
  - GP/clinics/hospitals/community support
- What wider support do you think should be available for people suffering with Long Covid
  - Functional/economic/physical/mental health/wellbeing

**We have talked a bit about your personal experience of Long Covid, it would be helpful now to get your thoughts on the wider discussion of Long Covid in the media, politics and research**

**7. General views on Long Covid**

**Are you aware of how Long Covid was identified?**

**What do you think about the name ‘Long Covid’?**

- Does it cover what you are experiencing?
- Do you think there should be a different name?
- What are your thoughts on the name Post-Covid syndrome?

**What do you think about Long Covid being compared to other illnesses e.g. ME/CFS**

- In what ways do you think this is helpful/unhelpful?

**What challenges are you aware of** **around Long Covid patients gaining access to treatment?**

**What others debates or discussions are you aware of around Long Covid?**

**8. Wrap up**

**Is there anything we’ve not talked about that you think is important or that you would like us to know?**

*Thank you for your time*
