# Supplementary material for: Awareness and perceptions of Long COVID among people in the REACT programme: Early insights from a pilot interview study
Source: PLoS One. 2023 Jan 26;18(1):e0280943. doi: 10.1371/journal.pone.0280943 (PMC9879384; doi:10.1371/journal.pone.0280943)
Supplement: S2 Appendix — (DOCX) [file pone.0280943.s003.docx]

**S2 Appendix 2. How the pilot study informs our main qualitative study.**

| Pilot study | Approach in main study |
| --- | --- |
| This pilot was based on a small sample of participants attending the LC recruitment clinic. | Recruiting up to 60 participants from a larger, more representative, sample within the REACT-LC cohort. |
| Our sample was limited in terms of the age, ethnicities and backgrounds of participants, and included people already in an existing research study which will have introduced participation biases. All participants were able to physically attend assessment clinics and we are likely to have not captured those experiencing more debilitating Long COVID symptoms. | Purposive sampling to ensure greater diversity of background and experience in our cohort. Primary sampling quotas (age, sex, ethnicity, pre-existing condition, deprivation) will be used. We will also monitor the location, symptom duration and time of Covid infection of participants. We will ensure we captures a range of severity (we will use ‘referred LC clinic’ or ‘referred to specialist’ as proxy sampling variables to give us an indication of those who’s symptoms required specialist treatment or support) |
| Participants in this study have had to consent at three time points (to join the REACT study, to join REACT-LC and to take part in the REACT-LC interview study) there are therefore more barriers to participation (such as receiving emails, reading study materials, finding time to opt in) than in a single-entry point study. | While we intend to do what we can to increase the ethnic diversity of the interview sample, we acknowledge that existing selection bias within the wider REACT-LC sample will determine who we are able to recruit.  If we are not able to achieve the desired diversity through are primary recruitment approach, we have contingency plans in place to access a wider sample, this may include sampling through the larger REACT-LC survey, our own community networks or using a snowball sampling approach. |
| For the pilot the main REACT-LC study information sheet was used. The provided information on the interviews alongside information on the clinical arm of the study rather than a dedicated sheet for interviews with those with persistent symptoms. Feedback on the main study information sheet suggested it was not visually appealing and the information about different aspects of the study were hard to find. | Study invitations and participant information sheets will focus on experiences of new or persistent symptoms rather than specifically using the term Long COVID. This will enable us to recruit participants who may not recognise or feel the term applies to them. During our interviews we will explore awareness and perceptions of the term Long COVID.  The participant information sheets have been developed with our public advisors to make the language and formatting more clear, accessible, and visually appealing, these will be made available to participants to view before they sign up, giving them time to read about the study. |
| The original topic guide, while developed with input from our public advisors with lived experience, was formed based on our early knowledge and awareness of the different manifestations of persistent symptoms of Covid-19. The pilot interviews helped advance our understanding and we became aware of gaps in the topics covered in the guide. We also found that the structure of the guide did not fit with the natural trajectory of the narratives shared by participants. | The topic guide will be adjusted to allow for more in-depth exploration into the lived experience of new and persistent symptoms following Covid-19 infection, including:  - Mapping change relative to participant baseline in 2019. In the new topic guide we will add questions about participant’s pre pandemic lifestyle. We will then use these examples throughout the interviews to prompt and probe participants on what changed and how their symptoms (and other contextual pandemic factors) have impacted these elements of their lifestyle.  - Asking about recovery. We will explore where people think they are in terms of ‘percentage recovered’, how long they expect to take to recover and at what point they would seek help if they have not recovered over time  - Asking about treatments or actions taken which participants think might have aided their recovery.  - Sensitively asking questions related to underexplored areas in which Long COVID might have had an impact such as sex and intimacy or changes to menstruation. |
| The broad range of topics covered by the guide and scoping nature of interviews resulted in largely descriptive findings. While each interview in the pilot was double coded by two researchers (EC and AL) the process of detection and categorisation was only carried out on a selected groups of codes related to participant perceptions of their own symptoms and of Long COVID as term and construct. | We intend to conduct more in depth and explanatory analysis on the data in the main qualitative study. We will move beyond categorisation of perspectives to higher order explanations, classifications, and themes.  We will undertake more in-depth and reflexive analysis. We anticipate that our approach will be informed by social science literature to allow us to draw on theory related to how illness is constructed, contested, and stigmatised when examining the evolving meaning of the term ‘Long COVID’. |
